# GeneDMRs: An R Package for Gene-Based Differentially Methylated Regions Analysis

**DOI:** 10.1089/cmb.2020.0081

**Published:** 2021-03-04

**Authors:** Xiao Wang, Dan Hao, Haja N. Kadarmideen

**Affiliations:** ^1^ Quantitative Genomics, Bioinformatics and Computational Biology Group, Department of Applied Mathematics and Computer Science, Technical University of Denmark, Kongens Lyngby, Denmark.; ^2^ College of Animal Science and Technology, Northwest A&F University, Yangling, China.; ^3^ Department of Molecular Biology and Genetics, Aarhus University, Aarhus C, Denmark.

**Keywords:** differentially methylated regions, DNA methylation, gene-based regions, geneDMRs, R package

## Abstract

DNA methylation in gene or gene body could influence gene transcription. Moreover, methylation in gene regions along with CpG island regions could modulate the transcription to undetectable gene expression levels. Therefore, it is necessary to investigate the methylation levels within the gene, gene body, CpG island regions, and their overlapped regions and then identify the gene-based differentially methylated regions (GeneDMRs). In this study, R package *GeneDMRs* aims to facilitate computing gene-based methylation rate using next-generation sequencing-based methylome data. The user-friendly *GeneDMRs* package is presented to analyze the methylation levels in each gene/promoter/exon/intron/CpG island/CpG island shore or each overlapped region (e.g., gene-CpG island/promoter-CpG island/exon-CpG island/intron-CpG island/gene-CpG island shore/promoter-CpG island shore/exon-CpG island shore/intron-CpG island shore). *GeneDMRs* can also interpret complex interplays between methylation levels and gene expression differences or similarities across physiological conditions or disease states. We used the public reduced representation bisulfite sequencing data of mouse (GSE62392) for evaluating software and revealing novel biologically significant results to supplement the previous research. In addition, the whole-genome bisulfite sequencing data of cattle (GSE106538) given the much larger size was used for further evaluation.

## 1. Introduction

Generally, gene expression is restricted by DNA methylation. However, the presence of methylation in gene or gene body could result in promotion of gene transcription. Irizarry et al. (2009) revealed the correlation between substantial portion of DNA methylation sites and gene expression along the genome. DNA methylation in promoters usually restricts the genes in a long-term stabilization of repressed states, whereas most gene bodies are also extensively methylated in different status; therefore, methylation of such regions can be the potential therapeutic targets (Jones, 2012; Yang et al., 2014). CpG islands, regions of high density of DNA methylation of cytosine and guanine dinucleotides (CpGs), are playing the important roles in gene regulation and transcriptional repression (Goldberg et al., 2007). Moreover, the shore regions beyond CpG islands are also involved in modulating gene expression (Doi et al., 2009; Irizarry et al., 2009).

Identifying causal relationships via genotype–phenotype correlations is a substantial challenge, and many studies across life sciences try to integrate multi-omics data sets in that effort (Suravajhala et al., 2016). Recently, one of the largest genetic study investigated global gene expression and DNA methylation patterns in 265 human skeletal muscle biopsies from the FUSION study with >7 million genetic variants. This integrated approach led to potential causal mechanisms for eight physiological traits: height, waist, weight, waist–hip ratio, body mass index, fasting serum insulin, fasting plasma glucose, and type 2 diabetes (Taylor et al., 2019). This underlines the importance of having gene-based methylation rates to integrate with differential expression or co-expression across physiological and phenotypic or disease states.

Studying DNA methylation patterns in biological samples using next-generation sequencing (NGS) methods is becoming increasingly common. There are several tools available to detect differentially methylated cytosine (DMC) [e.g., R package *IMA* (Wang et al., 2012), *MethylKit* (Akalin et al., 2012)] or differentially methylated region (DMR) [e.g., R package *COHCAP* (Warden et al., 2013), *ELMER* (Silva et al., 2018), *MethylMix* (Gevaert, 2015; Cedoz et al., 2018), *Minfi* (Aryee et al., 2014), *MIRA* (Lawson et al., 2018), *RnBeads* (Assenov et al., 2014; Müller et al., 2019)]. These packages mainly focus on specific differentially methylated regions such as genes (DMGs) from cancer data set (Gevaert, 2015; Cedoz et al., 2018) or only promoters (DMPs) (Assenov et al., 2014; Müller et al., 2019). However, detections of DMRs based on gene body features associated with CpG islands are scarce, such as DMRs in all exons (DMEs) and introns (DMIs) or a specific exon and intron.

To the best of our knowledge, there are no tools that detect the DMP/DME/DMI/DMG associated with CpG islands/CpG island shores. We present here a user-friendly R package *GeneDMRs* (gene-based differentially methylated regions; https://github.com/xiaowangCN/GeneDMRs) to facilitate computing gene-based methylation rate using NGS-based methylome data. *GeneDMRs* analyzes the methylation levels in each gene/promoter/exon/intron/CpG island/CpG island shore or each overlapped region (e.g., gene/promoter/exon/intron CpG island and gene/promoter/exon/intron CpG island shore). We evaluated *GeneDMRs* package using the publicly available reduced representation bisulfite sequencing (RRBS) data from mouse (GSE62392) and pig (GSE129385), and whole-genome bisulfite sequencing (WGBS) data from cattle (GSE106538).

## 2. Materials and Methods

### 2.1. Data structure in DNA methylation

Genome-wide DNA methylation analysis is mainly based on three approaches, that is, enzyme digestion, affinity enrichment, and bisulfite conversion (Laird, 2010). WGBS aims to find the whole methylome (Frommer et al., 1992), whereas RRBS primarily focuses on the enrichment of CpG-rich regions by recognizing the *CmCGG* site after restriction enzyme *MspI* digestion (Meissner et al., 2005), but both techniques rely on bisulfite conversion. From WGBS or RRBS data, cytosine site information (e.g., chromosome and position) and its methylation status can be obtained using available bioinformatics tools. *GeneDMRs* package can directly use the resulting methylation *coverage* file (i.e., *.bismark.cov*) from *Bismark* software (Krueger and Andrews, 2011) or similar file with chromosome, start position, end position, methylation percentage, number of methylated read, and number of unmethylated read (five or six columns). With such data set, we provide below the statistical framework to compute gene-based methylation rate.

### 2.2. Gene-based DMRs and analysis workflow

**FIG. 1.**
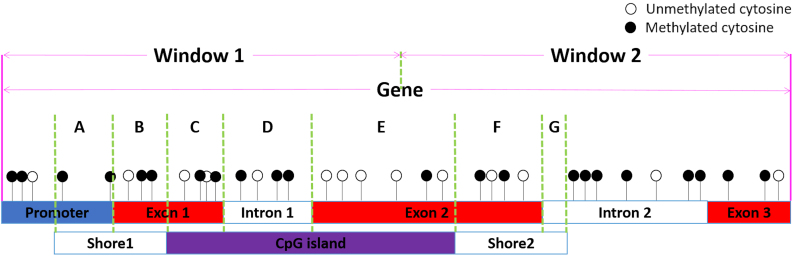
The analyzed targets in the *GeneDMRs* package including widows, genes (promoters, exons, introns), CpG islands (CpGis, Shores), and the overlapped feature regions [e.g., **(A)** Promoter-Shore1, **(B)** Exon1-Shore1, **(C)** Exon1-CpGi, **(D)** Intron1-CpGi, **(E)** Exon2-CpGi, **(F)** Exon2-Shore2, **(A** + **B)** Gene-Shore1 **(C** + **D** + **E)** Gene-CpGi, **(F + G)** Gene-Shore2]. *GeneDMRs,* gene-based differentially methylated regions.

The gene-based regions could be divided into windows, genes, promoters, exons, introns, CpG islands, and CpG island shores and their overlapped feature regions including gene-CpG islands, gene-CpG island shores, promoter-CpG islands, promoter-CpG island shores, exon-CpG islands, exon-CpG island shores, intron-CpG islands, and intron-CpG island shores (Fig. 1).

The methylation mean of a cytosine site by weighting for one group (a case or control) is calculated by Equation (1):

(1) $$\frac{MR_{i}}{\sum_{i=1}^{n}TR_{i}},$$

where $MR_{i}$ and $TR_{i}$ are the methylated and total read numbers at a given cytosine site of individual *i*, and *n* is the total number of individuals in one group.

**FIG. 2.**
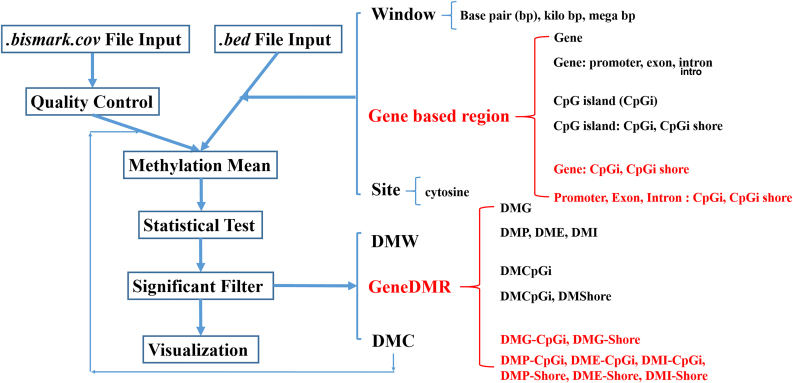
Overall workflow of *GeneDMRs* package.

For a window/gene (promoter, exon, intron)/CpGi/other overlapped region (Fig. 1) of one group, the methylation mean by weighting is calculated by Equation (2):

(2) $$\frac{\sum_{j=1}^{m}MR_{ij}}{\sum_{i=1}^{n}\sum_{j=1}^{m}TR_{ij}},$$

where $MR_{ij}$ and $TR_{ij}$ are the methylated and total read numbers of the involved cytosine/DMC *j* at a given gene/CpGi/other region of individual *i*, *m* is the total number of cytosines/DMCs involved in this region, and *n* is the total individual number of one group. For the target region, the cytosine/DMC within the region is chosen for the methylation mean calculation of each group. Here, the DMCs refer to the DMC sites after Significant_filter(siteall_Qvalue, qvalue = 0.01, methdiff = 0.05). Thus, if the users want to use DMCs for methylation mean, they should filter out the DMCs at first (Fig. 2). This step was also used in our previous study for methylation difference calculation to discover hyper- and hypomethylated DMGs (Wang and Kadarmideen, 2019a).

Logistic regression model was used to fit methylation levels with the different groups following the method of R package *MethylKit* (Akalin et al., 2012):

(3) $$\ln\left(\frac{\pi_{i}}{1-\pi_{i}}\right)=u+\beta T_{i},$$

where πi is the methylation mean of a cytosine calculated by Equation (1) or the methylation mean of a window/gene (promoter, exon, intron)/CpGi/other overlapped region calculated by Equation (2), *u* is the intercept, and *T_i_* is the group indicator.

More categorical variables can also be incorporated in this model as the additional covariates by Logic_regression(covariates = NULL). Chi-squared (*χ*^2^) test was used to determine the statistical significance of methylation differences among different groups and then to achieve the *p*-values. To account for multiple hypothesis testing, *p*-values of the analyzed cytosines or windows/genes (promoters, exons, introns)/CpGis/other overlapped regions can be adjusted to *Q*-values by various methods, for example, “bonferroni,” “holm” (Holm, 1979), “hochberg” (Hochberg, 1988), “hommel” (Hommel, 1988), “BH” (Hochberg, 1995), “fdr” (Hochberg, 1995), and “BY” (Benjamini and Yekutieli, 2001).

Differentially methylated windows or gene-based DMRs or DMCs (Fig. 2) are mainly filtered by *Q*-values and methylation level differences between two groups, for example, Significant_filter(qvalue = 0.01, methdiff = 0.05). The methylation difference can be calculated in Logic_regression(diffgroup = c(“group1”, “group2”)) by selecting any two groups. The DMGs can be defined as the hyper-/hypomethylated genes when the methylation differences are positive/negative after case–control comparisons (e.g., group2–group1). Therefore, DMRs for specific regions are detected, such as genes (DMGs), promoters (DMPs), exons (DMEs), introns (DMIs), CpG islands (DMCpGis), CpG island shores (DMShores), and the overlapped regions such as gene-CpG islands (DMG-CpGis), gene-CpG island shores (DMG-Shores), promoter-CpG islands (DMP-CpGis), promoter-CpG island shores (DMP-Shores), exon-CpG islands (DME-CpGis), exon-CpG island shores (DME-Shores), intron-CpG islands (DMI-CpGis), and intron-CpG island shores (DMI-Shores; Fig. 2). Furthermore, the ordinal positions of exons and introns were identified for each gene, which can be used in the further analysis, for example, the correlations of methylation levels between all promoters and all first exons (Wang and Kadarmideen, 2020). The overall workflow of *GeneDMRs* package includes file input, quality control (QC), methylation mean calculation, statistical test, significant filter, and results visualization (Fig. 2).

### 2.3. Application to real data

The RRBS data for testing the package were download from Gene Expression Omnibus (GEO) with the accession number GSE62392 (https://www.ncbi.nlm.nih.gov/geo/query/acc.cgi?acc=GSE62392). The downloaded data were originally generated from RRBS of sorted common myeloid progenitor (CMP) populations that were isolated from three pools of G0 as control group and two pools of G5 as case group of mice samples (Colla et al., 2015). Mouse data here are used as an example, and *GeneDMRs* package is applicable to all species. We applied several pre and postmapping QC to these data as follows. Adapters and reads less than 20 bases long of RRBS data were trimmed by *Trimmomatic* software (version 0.36) (Bolger et al., 2014). The clean reads were mapped to the mice reference genome by *Bowtie 2* software (version 2.3.3.1) (Langmead and Salzberg, 2012). The house mouse (*Mus musculus*) reference genome (mm10) used in this study was downloaded from the University of California Santa Cruz (UCSC) website (http://hgdownload.soe.ucsc.edu/goldenPath/mm10/bigZips/mm10.2bit). The *.2bit* file was subsequently converted to *.fasta* file by *twoBitToFa* software (http://hgdownload.cse.ucsc.edu/admin/exe/linux.x86_64/twoBitToFa). Finally, read coverages of detected methylated or unmethylated cytosine sites and their methylation percentages were obtained by using *Bismark* software (version 0.19.0) (Krueger and Andrews, 2011).

### 2.4. Input and QC

The resulting methylation *coverage* files from *Bismark* software of five mouse RRBS data were directly used as input in *GeneDMRs* package. The public mouse (mm10) *bed* file (i.e., *.bed*) for Reference Sequence (refseq) and CpG island (cpgi) database was downloaded from the UCSC web site (http://genome.ucsc.edu/cgi-bin/hgTables). RefSeq and CpG island *bed* files were used as input files in *GeneDMRs* package, which then can output four files (inputrefseqfile, inputcpgifile, inputgenebodyfile, and inputcpgifeaturefile) by altering the *feature* parameter in the file reading function, for example, Bedfile_read(feature = TRUE/FALSE). Bedfile_read() function divides each gene of refseq *bed* file into four gene body features (i.e., promoters, exons, introns, and TSSes) and each CpG island of cpgi *bed* file into two CpG island features (i.e., CpG islands and CpG island shores) based on R package *genomation* (Akalin et al., 2015). Moreover, Bedfile_read() function annotates specific gene to each promoter. If the strand of one promoter is “+”/“−,” the middle position of this promoter will be the start/end position of the matched specific gene. However, for the specific genes with more than one National Center for Biotechnology Information (NCBI) ID, *GeneDMRs* package will choose the first one. For example, the adenosine A1 receptor gene (*Adora1*) has four NCBI IDs (i.e., NM_001291930, NM_001282945, NM_001039510, and NM_001008533) and only the first ID (NM_001291930) will be chosen.

When a polymerase chain reaction experiment suffers from duplication bias, some clonal reads will impair accurate determination of methylation (Akalin et al., 2012). In addition, lower read coverages (e.g., lower than 10) will cause the biases for methylation percentage calculation (Wang and Kadarmideen, 2019b). Therefore, cytosines with a percentile of read coverage higher than the 99.9th and read coverages lower than 10 were discarded for the qualified reads by Methfile_QC(high_quantile = 99.9, low_coveragenum = 10).

### 2.5. Biological enrichment for the DMGs

The enrichments of gene ontology (GO) terms and pathways for DMGs were analyzed and visualized by Enrich_plot(enrichterm = c(“GO”, “pathway”), category = TRUE/FALSE) based on R package *clusterProfiler* (Yu et al., 2012). If the category = TRUE, the enrichment will display in hypermethylated and hypomethylated categories. In addition, the differentially expressed genes (DEGs) with Log fold change (LogFC) information can also be used in Enrich_plot(expressionfile_significant = NULL), then the visualized enrichment will be in four categories that are hyper-/hypomethylated and up-/downregulated genes. The up-/downregulated DEG can be defined when the LogFC is positive/negative. Here, we use the previous results for multiple-category enrichments that are downregulated and upregulated genes in G4/G5 compared with G0 CMP (fdr <0.05) of mice samples (https://ars.els-cdn.com/content/image/1-s2.0-S1535610815001403-mmc2.xlsx) (Colla et al., 2015).

## 3. Results and Discussion

### 3.1. Comparisons of different R packages for methylation analysis

Currently, a series of R packages can analyze methylation data to detect DMCs or DMRs (Table 1). Most of them are not, however, completely focusing on the regions in genes or within gene bodies or CpG islands, and hence, *GeneDMRs* package could be a complementary tool. As shown in Table 1, *ELMER* package reconstructs altered gene regulatory network by combining enhancer methylation and gene expression (Silva et al., 2018). *IMA* (Wang et al., 2012) and *MethylKit* (Akalin et al., 2012) aim at genome-wide cytosine sites analysis for BeadChip and RRBS data, respectively. Generally, *COHCAP*, *methyAnalysis*, *MethylationArrayAnalysis*, and *Minfi* are packages for specific purposes, where *COHCAP* refines the region boundaries for the consistent methylation patterns through a clustering step (Warden et al., 2013), *methyAnalysis* applies CpG island information to visualize in the heat map plot, and *Minfi* can find the hypomethylation blocks (Jaffe et al., 2012; Aryee et al., 2014). If considering methylated genes, *MethylMix* package mainly focuses on identifying disease specific hypo- and hypermethylated genes, and it defines the methylation difference of a methylation state with the normal methylation state (Gevaert, 2015; Cedoz et al., 2018), whereas *RnBeads* package could consider the gene, gene promoter, CpG island, and genomic tiling regions (Assenov et al., 2014; Müller et al., 2019). Overall, none of these R packages works for gene components, but *GeneDMRs* package is extended to exon and intron regions, and their interactions with CpG island features.

**Table 1.** Comparisons of Different R Packages for Methylation Analysis

R package	Target	Analysis feature	Issued time
*COHCAP* (Warden et al., 2013)	Site and region of differential methylation	Identify differentially methylated CpG islands and show the consistent methylation patterns among CpG sites by refinement of region boundaries through a clustering step	2013
*ELMER* (Silva et al., 2018)	DMR	Reconstruct altered GRN by combining enhancer methylation and gene expression	2018
*IMA* (Wang et al., 2012)	Site-level and region-level methylation	Summarization for individual site as well as annotated region	2012
*methyAnalysis*	DMR	Chromosome location-based DNA methylation analysis and heat map plot with CpG island	2018
*MethylationArrayAnalysis*	Probe-wise differential methylation and DMR	Differential variability analysis, estimating cell-type composition and gene ontology testing	2019
*MethylKit* (Akalin et al., 2012)	Base or region of DNA methylation	Functions for clustering, sample quality visualization, differential methylation analysis, and annotation feature	2012
*MethylMix* (Gevaert, 2015)/*MethylMix 2.0* (Cedoz et al., 2018)	DMR of gene	Automate the construction of DNA methylation and gene expression data set from TCGA	2015/2018
*Minfi* (Jaffe et al., 2012; Aryee et al., 2014)	DMP and bump hunting of DMR	Block finding to identify hypomethylation block	2014
*MIRA* (Lawson et al., 2018)	DMR	Take advantage of genome-scale DNA methylation data to assess regulatory activity	2018
*RnBeads* (Assenov et al., 2014)/*RnBeads 2.0* (Müller et al., 2019)	DMR of gene/promoter/CpG island	DNA methylation-based prediction of age and sex; LOLA-based region set enrichment analysis for biological interpretation	2014/2019

DMP, differentially methylated position; DMR, differentially methylated region; GRN, gene regulatory network; TCGA, The Cancer Genome Atlas.

**FIG. 3.**
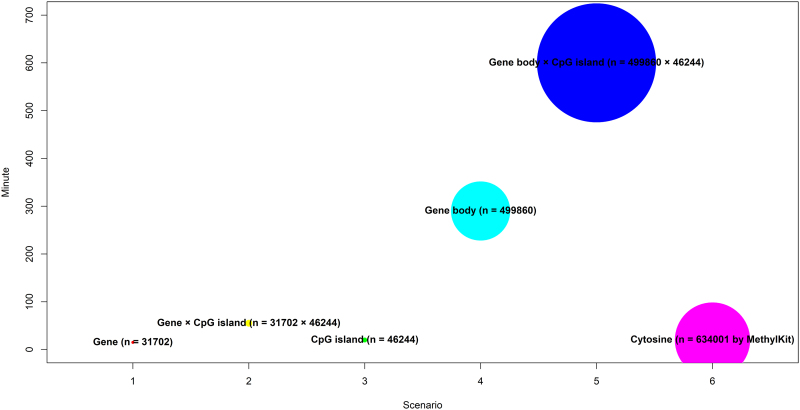
The performance of *GeneDMRs* package.

The performance of the package was tested in a personal computer (CPU: 2.70 GHz, RAM: 8.00 GB) comparing with *MethylKit* package (Akalin et al., 2012). For all reference genes (*n* = 31,702) of mouse RRBS data with around 0.7 million analyzed CpG sites, *GeneDMRs* package took around 15 minutes while gene body interacted with CpG island required the longest time; thus, the performance of the package is generally related to the number of analyzed targets (Fig. 3). In addition, we applied another two data sets given the much larger size using the same parameters as mouse data set for performance test. One was download from GEO with the accession number GSE129385 (https://www.ncbi.nlm.nih.gov/geo/query/acc.cgi?acc=GSE129385) that is also RRBS sequencing data from nine porcine testis samples (Wang and Kadarmideen, 2019a, 2020). Another one was downloaded from GEO with the accession number GSE106538 (https://www.ncbi.nlm.nih.gov/geo/query/acc.cgi?acc=GSE106538) that is WGBS sequencing data from four bovine sperm samples (Zhou et al., 2018; Fang et al., 2019). For all reference genes (*n* = 4475) and all gene bodies (*n* = 77,022) of porcine RRBS data with around 1 million analyzed CpG sites, *GeneDMRs* package completed the whole DMR detections in around 1 minute and 1 hour, respectively. While using bovine WGBS data for all reference genes (*n* = 14,391) analysis with around 7 million sites, it only needed 10 minutes. When increasing the analyzed targets for all gene bodies (*n* = 279,903), the analyzing time increased to 3 hours. However, keeping all the raw sites ∼50 million, 6 hours or longer time were required for all reference genes or gene bodies.

### 3.2. DMG-based regions and cytosine sites

Five methylation *coverage* files from *Bismark* software were used in *GeneDMRs* package, and their statistical summary is listed in Supplementary Table S1. The *GeneDMRs* package will automatically read the files with the file name such as “1_1,” “1_2,” “2_1,” and “2_2” for group and replicate numbers. The methylation patterns of all genes and DMGs in different CpG island regions by Group_cpgfeature_boxplot() and Genebody_cpgfeature_boxplot() are shown in Supplementary Figure S1. Results suggest that the methylation levels of DMGs were higher than before, and they are the same of CpG islands lower than shores (Supplementary Fig. S1). All data sets for genes (regiongeneall_Qvalue), genes with CpG island features (regiongeneall_cpgfeature_Qvalue), gene bodies with CpG island features (genefeatureall_cpgfeature_Qvalue), and cytosine sites (genefeatureall_cpgfeature_Qvalue) are listed in Supplementary Files S1–S4, respectively.

**FIG. 4.**
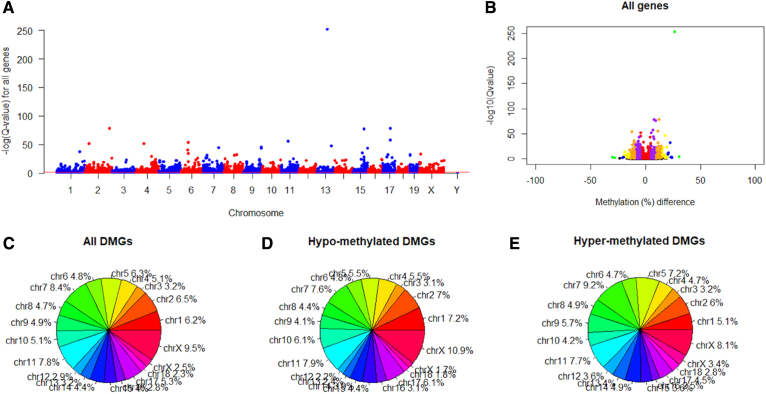
**(A)** Manhattan plots for all genes. The red line indicates the significant level of *Q*-value <0.01. **(B)** Methylation differences in all genes. Plots showing red, purple, orange, yellow, blue, and green colors indicate genes with a *Q*-value <0.01 and methylation difference (%) greater than 0, 5, 10, 15, 20, and 25, respectively. **(C)**–**(E)** Percentages of all, hypomethylated, and hypermethylated DMGs in different chromosomes, respectively. DMGs, differentially methylated genes.

The methylation difference of all cytosine sites involved in the gene was centralized to a mean, so statistical power seemed be lower than before (Fig. 4 and Supplementary Fig. S2). In addition, *GeneDMRs* package can detect various gene body regions (e.g., promoter, exon, and intron), CpG island regions (e.g., CpGi and shore regions), and their overlapped regions by Methmean_region(cpgifeaturefile = inputcpgifeaturefile/NULL, featureid = “c(“chr1”,“chr2”)/all/alls”, featurename = c(“promoters”,“exons”,“introns”,“TSSes”)/c(“CpGisland”, “Shores”)).

According to these results, we found that *DNMT3A* was a hypomethylated gene (NM_001271753), but the gene and one intron interacted in both CpG island and shore features were in hypermethylation status when G5 CMP was compared with G0 CMP (Supplementary Files S1–S3). Therefore, *GeneDMRs* package can accurately find significantly and biologically methylated gene body and CpG island regions along the whole genome and supplement the previous research (Colla et al., 2015).

**FIG. 5.**
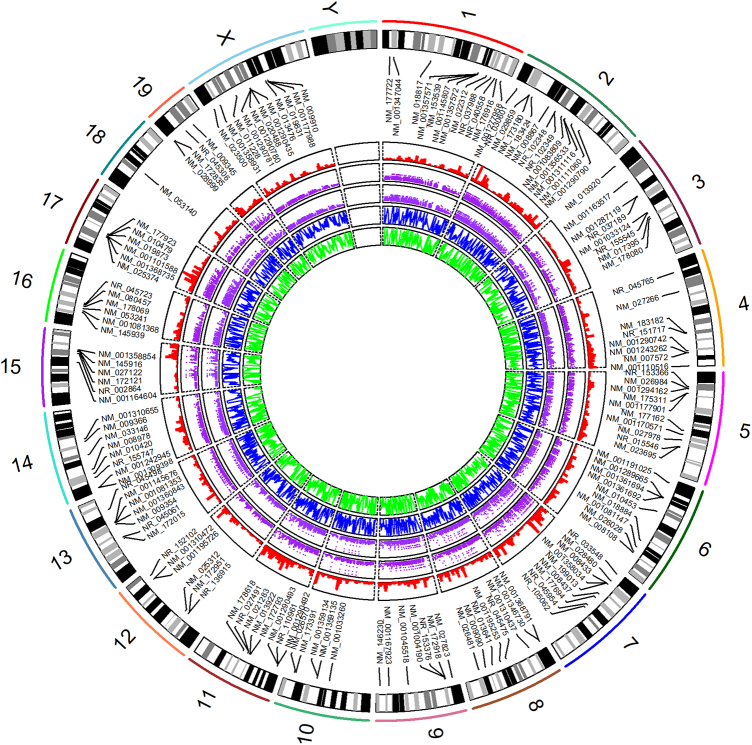
Circular graph of the global methylation levels. From the outermost track to innermost circle, the circles indicate genome chromosomes (i.e., mouse), DMGs, gene densities, CpG island densities, CpG island shore densities, and methylation levels. The densities and methylation levels were calculated by 1,000,000 bp windows, that is, Window_divide(windowbp = 1000000).

If we only use the DMCs to recalculate the methylation mean by replacing the cytosine sites, that is, DMC_methfile_QC(inputmethfile_QC, siteall_significant), the methylation difference will be more obvious than before (Supplementary Fig. S3). The global DMC-based methylation levels (Fig. 5) can be realized by Circos_plot(inputcytofile, inputmethfile_QC, inputrefseqfile, inputcpgifeaturefile) based R package *RCircos* (Zhang et al., 2013).

### 3.3. Biological enrichment for DMGs

**FIG. 6.**
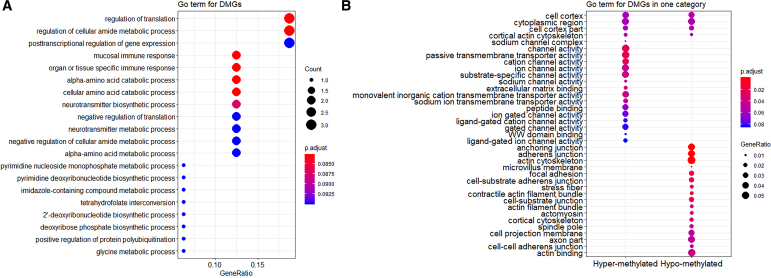

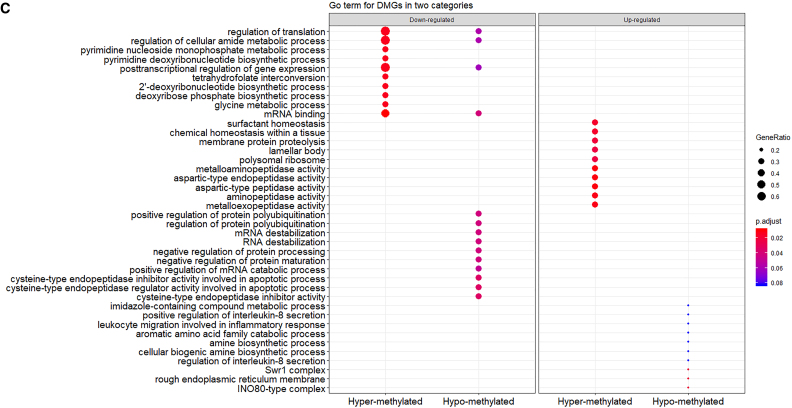
GO term enrichments. **(A)** GO terms without category. **(B)** GO terms with one category of hyper-/hypomethylated genes. **(C)** GO terms with two categories of hyper-/hypomethylated and up-/downregulated genes. GO, gene ontology.

The enrichments for groups, GO terms, and pathways can be analyzed and visualized with/without categories following R packages *clusterProfiler* (Yu et al., 2012). For example, the GO terms can be visualized in no/one/two categories (Fig. 6) by incorporating hyper-/hypomethylated and up-/downregulated gene information. Thus, based on the DMGs and enrichments for GO term and pathway, *GeneDMRs* package can help to detect the specific significant regions, reveal the biological mechanism, and enhance the previous studies that methylation pattern changes in specific regions were involved in causing diseases (Colla et al., 2015).

## 4. Summary

Currently, there is no easy-to-use R package that could compute methylation levels at gene-based level. *GeneDMRs*, a user-friendly R package, can facilitate computing gene-based methylation rate using NGS-based methylome data. This package aims to analyze the methylation levels in gene/promoter/exon/intron/CpG island/CpG island shore and their overlapped regions. Then, the differentially hyper-/hypomethylated genes can be visualized for enrichments of GO terms and pathways and reveal the biological mechanism accordingly. Such gene-based methylation analyses contribute to interpreting complex interplay between methylation levels and gene expression differences or similarities across physiological conditions or disease states.

## Availability and Implementation

GeneDMRs is freely available at https://github.com/xiaowangCN/GeneDMRs

## Supplementary Material

Supplemental data

Supplemental data

Supplemental data

Supplemental data

Supplemental data

Supplemental data

Supplemental data

Supplemental data
