# Supplementary material for: GeneDMRs: An R Package for Gene-Based Differentially Methylated Regions Analysis
Source: J Comput Biol. 2021 Mar 4;28(3):304–16. doi: 10.1089/cmb.2020.0081 (PMC7994424; doi:10.1089/cmb.2020.0081)
Supplement: Supplemental data [file Supp_Table1.docx]

Supplementary table 1. Statistical summary of data source.

| Sample | Name in *GeneDMRs* package | Clean read pair | Uniquely mapping efficiency | Total cytosine number | Cytosine methylation rate in CpG context | Cytosine methylation rate in CHG context | Cytosine methylation rate in CHH context |
| --- | --- | --- | --- | --- | --- | --- | --- |
| G0-CMP1 | 1_1 | 30,015,396 | 62.1% | 201,134,947 | 21.6% | 0.3% | 0.3% |
| G0-CMP2 | 1_2 | 30,843,564 | 63.0% | 205,241,712 | 24.4% | 0.4% | 0.3% |
| G0-CMP3 | 1_3 | 32,558,450 | 62.4% | 215,559,294 | 23.1% | 0.4% | 0.3% |
| G5-CMP1 | 2_1 | 28,780,302 | 61.4% | 193,333,818 | 19.9% | 0.3% | 0.3% |
| G5-CMP2 | 2_2 | 31,798,819 | 60.7% | 204,493,356 | 24.9% | 0.3% | 0.3% |
