# Supplementary material for: GeneDMRs: An R Package for Gene-Based Differentially Methylated Regions Analysis
Source: J Comput Biol. 2021 Mar 4;28(3):304–16. doi: 10.1089/cmb.2020.0081 (PMC7994424; doi:10.1089/cmb.2020.0081)
Supplement: Supplemental data [file Supp_Fig1.docx]

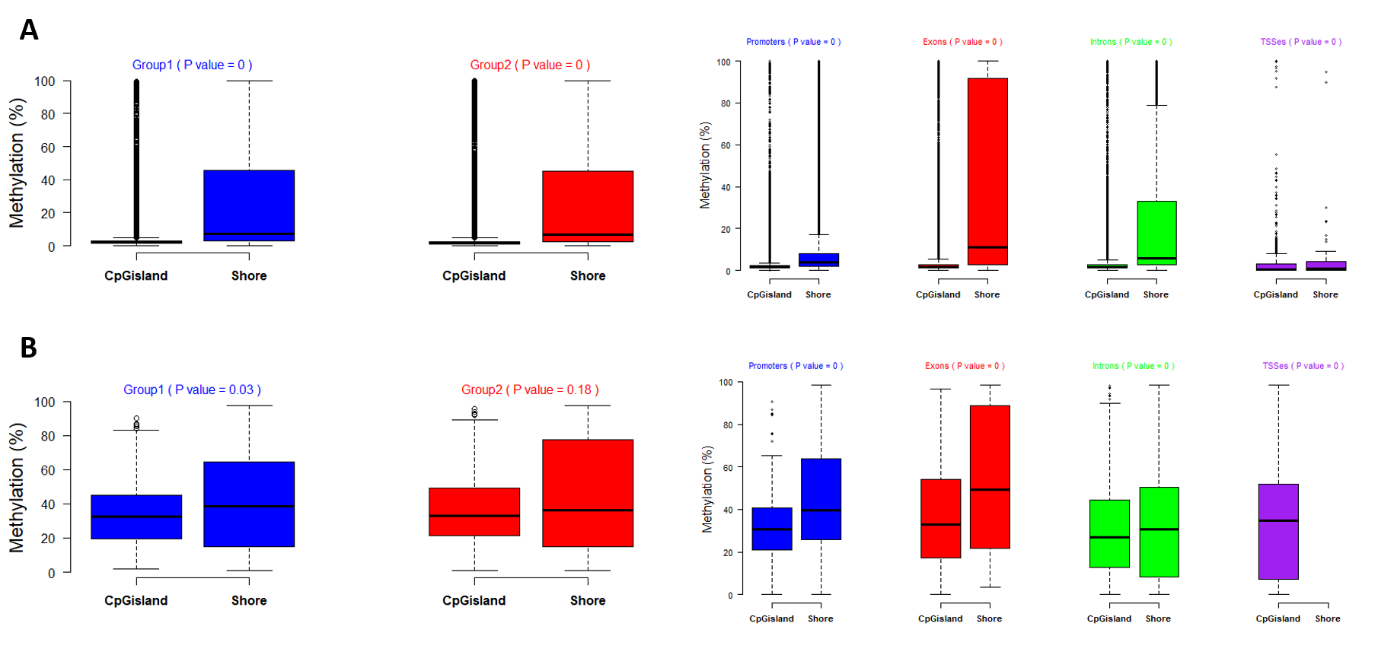


Supplementary figure 1. (**A**) Methylation patterns of all genes/cytosine sites for different groups and gene bodies in different CpG island regions. (**B**) Methylation patterns of all DMGs/DMCs for different groups and gene bodies in different CpG island regions. Note: P value is calculated by the methylation comparison between CpG island and CpG island shore with Student’s t-tests.
