# Supplementary material for: GeneDMRs: An R Package for Gene-Based Differentially Methylated Regions Analysis
Source: J Comput Biol. 2021 Mar 4;28(3):304–16. doi: 10.1089/cmb.2020.0081 (PMC7994424; doi:10.1089/cmb.2020.0081)
Supplement: Supplemental data [file Supp_Fig2.docx]

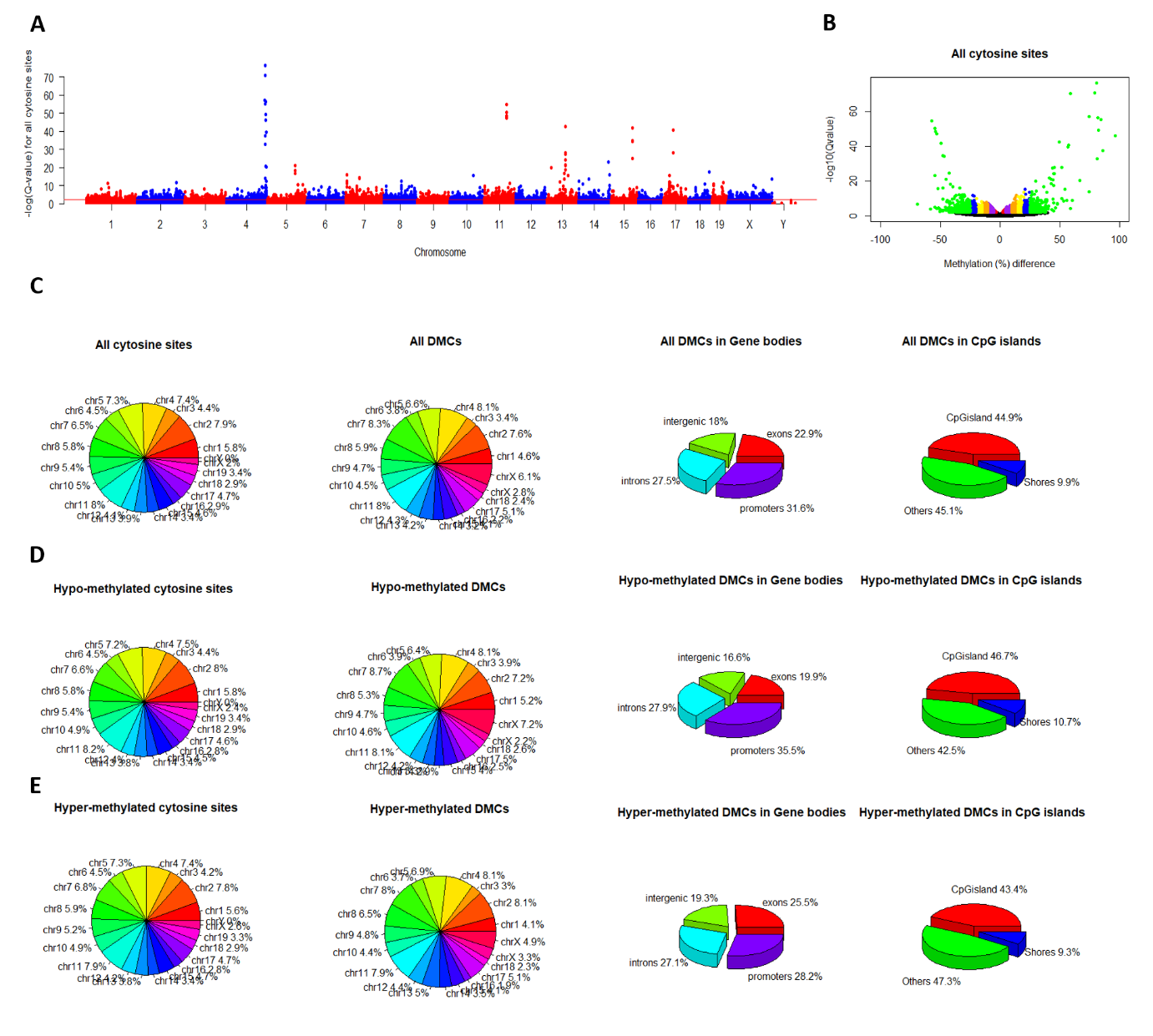


Supplementary figure 2. (**A**) Manhattan plots for all cytosine sites. Note: The red line indicates the significant level of Q-value < 0.01. (**B**) Methylation differences in all cytosine sites. Note: Plots showing red, purple, orange, yellow, blue and green colors indicate genes with a Q-value less than 0.01 and methylation difference (%) greater than 0, 5, 10, 15, 20 and 25, respectively. (**C**), (**D**) and (**E**) Percentages of all, hypo-methylated and hyper-methylated cytosine sites/DMCs in different chromosomes/gene bodies/CpG islands, respectively.
