# Supplementary material for: GeneDMRs: An R Package for Gene-Based Differentially Methylated Regions Analysis
Source: J Comput Biol. 2021 Mar 4;28(3):304–16. doi: 10.1089/cmb.2020.0081 (PMC7994424; doi:10.1089/cmb.2020.0081)
Supplement: Supplemental data [file Supp_Fig3.docx]

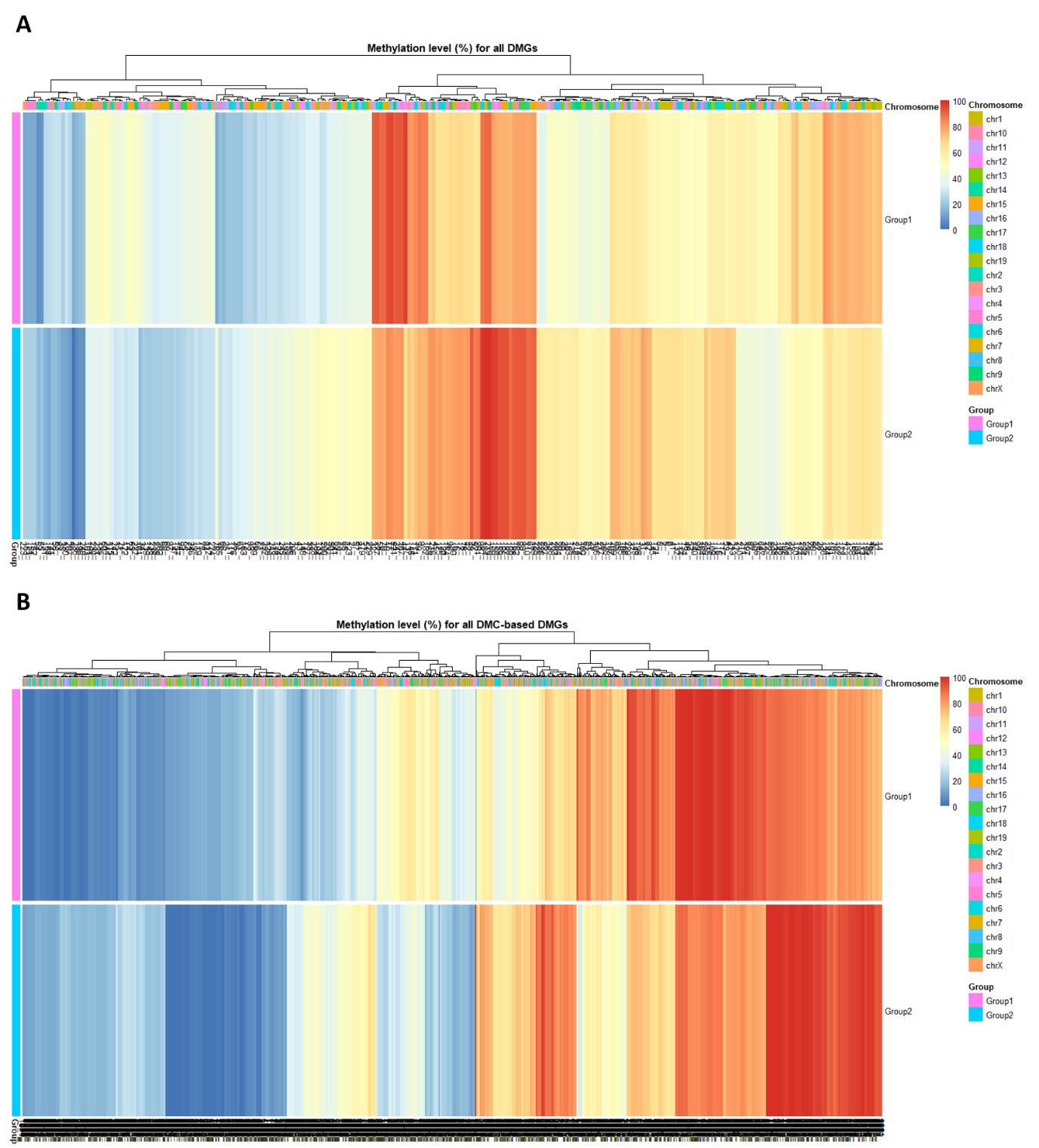


Supplementary figure 3. (**A**) Heat map cluster for methylation levels of all DMGs (n = 246). (**B**) Heat map cluster for methylation levels of all DMC-based DMGs (n = 2022). Note: DMGs and DMC-based DMGs were filter by Significant_filter(qvalue = 0.01, methdiff = 0.1).
